# Dietary patterns associated with the incidence of hypertension among adult Japanese males: application of machine learning to a cohort study

**DOI:** 10.1007/s00394-024-03342-w

**Published:** 2024-02-25

**Authors:** Longfei Li, Haruki Momma, Haili Chen, Saida Salima Nawrin, Yidan Xu, Hitoshi Inada, Ryoichi Nagatomi

**Affiliations:** 1 School of Physical Education and Health, Heze University, 2269 University Road, Mudan District, Heze, 274-015 Shandong China; 2 Department of Medicine and Science in Sports and Exercise, Tohoku University Graduate School of Medicine, 2-1 Seiryo-Machi, Aoba-ku, Sendai, Miyagi 980-8575 Japan; 3 Division of Biomedical Engineering for Health & Welfare, Tohoku University Graduate School of Biomedical Engineering, 6-6-12, Aramaki Aza Aoba Aoba-ku, Sendai, Miyagi 980-8579 Japan; 4 Department of Developmental Neuroscience, Tohoku University Graduate School of Medicine, 2-1 Seiryo-Machi, Aoba-ku, Sendai, Miyagi 980-8575 Japan; 5 Department of Biochemistry and Cellular Biology, National Center of Neurology and Psychiatry, 4-1-1 Ogawa-Higashi, Kodaira, Tokyo, 187-8502 Japan

**Keywords:** Unsupervised machine learning, UMAP, K-means, Dietary patterns, Hypertension

## Abstract

**Purpose:**

The previous studies that examined the effectiveness of unsupervised machine learning methods versus traditional methods in assessing dietary patterns and their association with incident hypertension showed contradictory results. Consequently, our aim is to explore the correlation between the incidence of hypertension and overall dietary patterns that were extracted using unsupervised machine learning techniques.

**Methods:**

Data were obtained from Japanese male participants enrolled in a prospective cohort study between August 2008 and August 2010. A final dataset of 447 male participants was used for analysis. Dimension reduction using uniform manifold approximation and projection (UMAP) and subsequent K-means clustering was used to derive dietary patterns. In addition, multivariable logistic regression was used to evaluate the association between dietary patterns and the incidence of hypertension.

**Results:**

We identified four dietary patterns: ‘Low-protein/fiber High-sugar,’ ‘Dairy/vegetable-based,’ ‘Meat-based,’ and ‘Seafood and Alcohol.’ Compared with ‘Seafood and Alcohol’ as a reference, the protective dietary patterns for hypertension were ‘Dairy/vegetable-based’ (OR 0.39, 95% CI 0.19–0.80, *P* = 0.013) and the ‘Meat-based’ (OR 0.37, 95% CI 0.16–0.86, *P* = 0.022) after adjusting for potential confounding factors, including age, body mass index, smoking, education, physical activity, dyslipidemia, and diabetes. An age-matched sensitivity analysis confirmed this finding.

**Conclusion:**

This study finds that relative to the ‘Seafood and Alcohol’ pattern, the ‘Dairy/vegetable-based’ and ‘Meat-based’ dietary patterns are associated with a lower risk of hypertension among men.

**Supplementary Information:**

The online version contains supplementary material available at 10.1007/s00394-024-03342-w.

## Introduction

Hypertension has been recognized as a serious health risk [1], affecting a staggering number of over 1.1 billion individuals [2]. Especially uncontrolled hypertension is a risk factor for future cardiovascular diseases globally [3]. Patients with hypertension were estimated as 43 million in Japan [4–6]. Hypertension is a complex condition influenced by genetic and environmental factors, but environmental factors play a primary role in its development and progression [7, 8]. Therefore, mitigating the effects of environmental factors to minimize or delay the development of hypertension is a significant challenge in hypertension management and prevention policies.

Dietary factors have been intensively investigated as one of the environmental factors associated with the risk of hypertension [9, 10]. To simplify the situation, many studies have focused on the relationship between the risk of hypertension and particular foods or nutrients [11], particularly the effect of a single nutrient, food, or food group. However, this approach has several limitations because foods and nutrients are generally consumed with interactive or synergistic effects [12]. Some healthy dietary patterns (e.g., a healthy diet with the consumption of nuts and seeds, low-fat dairy products, vegetables, fruits, poultry, and fish; and an unhealthy diet with foods such as red meat, fast food, high-fat dairy products, refined grain, salty snacks, and drinks, etc.) are associated with a lower blood pressure and risk of hypertension [13–16]. Generally, a dietary pattern is a comprehensive variable that integrates the consumption of several food groups. For instance, the ‘fruit and milk’ pattern was associated with a lower prevalence of hypertension among adult Chinese men [14], and the traditional Japanese diets, which are characterized by high consumption of vegetables, seaweeds, mushrooms, potatoes, fruits, fish, and soybeans including tofu and miso, were significantly related to lower blood pressure [15, 17].

Furthermore, dietary behavior (e.g., more frequent meals at home, eating speed, or skipping breakfast) and cooking methods (e.g., boiling or frying) influence hypertension [18–21]. Food and taste preferences could also impact hypertension. For instance, it has been reported that the frequency of dairy product consumption is related to a risk of hypertension [19] and that reducing salt and seasoning consumption significantly improves blood pressure [20]. The combination of food intake, dietary behavior, and cooking methods has been shown to affect the disease risk more than any single nutrient [18, 22]. Therefore, a more holistic approach is needed to capture the dietary tendency or patterns, including food items, dietary behaviors, and cooking methods.

Two main approaches used to extract dietary patterns are hypothesis-driven and data-driven approaches [23–25]. The first approach aims to calculate a graded score or index based on recommended diets or dietary guidelines [26, 27]. However, the hypothesis-driven approaches have two limitations: (1) they do not consider the correlation structure between food frequency intake and dietary habits (dietary behavior and cooking methods), and (2) they reflect only the individual effects of each food or nutrient without considering how they interact with each other within a diet [28]. The data-driven approach, also known as the a posteriori or exploratory approach, involves extracting dietary patterns from the data [26]. This approach uses statistical exploratory procedures such as principal component analysis (PCA) [13, 16], cluster methods [29] and factor analysis [15], which are widely applied in nutritional epidemiology. PCA is a commonly used data-driven method to derive dietary patterns [30, 31]; however, despite its popularity in dietary pattern studies, it has several limitations. Although PCA has been used for the dimension reduction of multidimensional datasets, the procedure generally results in a loss of information [32]. Furthermore, the standard PCA cannot analyze nonlinear relationships [33] or categorical data reported as dietary habits, such as dietary behavior and cooking methods.

Unsupervised machine learning analysis is a recent procedure for data-driven approaches to identify dietary patterns [34]. This approach has recently been applied in some studies to reveal different dietary patterns [35, 36]. Unsupervised machine learning techniques for clustering and dimensionality reduction algorithms [37] can be used to overcome some PCA limitations and improve the results. Uniform manifold approximation and projection (UMAP) is a nonlinear technique capable of preserving the global structure of the data, making it well suited for use in unsupervised clustering applications [38]. Compared with PCA, UMAP has the advantage of handling categorical variables and preserving relationships between categorical variables and other features in the data [39]. UMAP makes it an attractive option for unsupervised clustering, where the goal is to identify patterns and relationships in the data without needing prior knowledge or labeled data. Therefore, combining food intake, dietary behavior, and cooking methods based on unsupervised technique analysis may be more suitable for deriving dietary patterns.

The first purpose of this study was to derive dietary patterns among Japanese men based on a brief-type self-administered diet history questionnaire (BDHQ) dataset using unsupervised machine learning. The second purpose was to examine the association between extracted clusters of dietary patterns and the incidence of hypertension to show the validity of the novel method for identifying dietary patterns. We show here how these UMAP-identified patterns are associated with incidence of hypertension among Japanese male population. Furthermore, in this study, we will explore how these identified dietary patterns are linked to the incidence of hypertension among the Japanese population. Our study would provide helpful insights into dietary interventions for the prevention of hypertension in Japanese males.

## Methods

### Participants and selection design

This study used data from the Oroshisho study, a longitudinal study of the lifestyle-related influences on chronic noncommunicable diseases and health status of 1,253 individual Japanese adult employees working at Sendai Oroshisho Center Sendai, conducted between 2008 and 2011. Details of the Oroshisho study have been described previously [40]. The protocol for the current study was approved by the Institutional Review Board of Tohoku University School of Medicine (Approval Number: 2019-1-394).

**Fig. 1:**
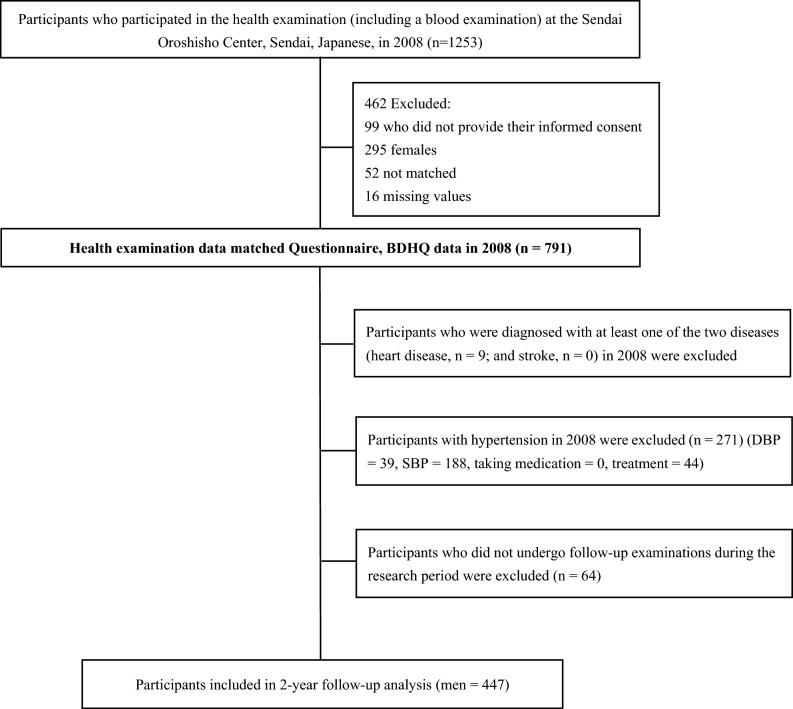
Flowchart of participants included in the analysis

The 2008 dataset was used as the baseline. The selection procedure for the participants is presented in Fig. 1. Briefly, we recruited 1,253 individuals at baseline who underwent an annual health examination. Our study employed data collected in 2008 (as a baseline) and 2010 (as a follow-up). Of the participants, 1154 provided written informed consent to participate in the study, resulting in a 92.1%. Female participants (*n* = 295) were also excluded from the analysis due to their sample size, low prevalence, and, especially, sex-related difference in the risk of hypertension, which was demonstrated by a recent meta-analyses [41]. Among the excluded participants, 68 individuals were removed from the analysis due to incomplete baseline data, including missing dietary information (*n* = 16) and incomplete health examination records (*n* = 52). Additionally, a total of 344 individuals were excluded from the analysis due to incomplete baseline data, which included the presence of heart disease (*n* = 9), systolic blood pressure ≥ 140 mmHg (*n* = 188), diastolic blood pressure ≥ 90 mmHg (*n* = 39), self-reported history of previously diagnosed hypertension or current medication for hypertension at the health examination (*n* = 44), and failure to undergo follow-up examinations during the study period (*n* = 64). We did not include data from 2011 because some participants may have changed their dietary habits due to the 2011 Great East Japan Earthquake in March 2011. It was possible that the study participants have chosen to eat less seafood than before, considering the risk of potential contamination of agricultural and fishery products due to the Fukushima nuclear plant accident. Finally, 447 male participants were included in the final analysis (Fig. 1).

### Blood pressure measurement and hypertension diagnosis

Systolic and diastolic blood pressures were measured in the upper right arm using an automatic device (Yamasu 605P; Kenzmedico, Saitama, Japan) in a sitting position according to our previous study [42]. According to the study protocol, a participant with a systolic blood pressure of > 150 mmHg or diastolic blood pressure of > 100 mmHg at the initial measurement was considered to undergo a second measurement. In such case, the blood pressure values of the second measurement were adopted. Hypertension was defined as a systolic blood pressure ≥ 140 mmHg, diastolic blood pressure ≥ 90 mmHg, self-reported history of hypertension, or use of medication for hypertension. Individuals without hypertension at baseline (2008) were considered to have incident hypertension when they met any of these conditions during the subsequent health checkups from August 2009 to August 2010.

### Analysis of dietary habits

Information about dietary habits was obtained through the BDHQ [43]. In the BDHQ, 79 items were inquiries about food intake of 58 different food items, 12 dietary behavior, and 9 cooking methods. For food intakes, the participants indicated their mean frequency of consumption in terms of the specified serving size by checking one of seven frequency categories ranging from ‘almost never’ to ‘two or more times/day.’ For alcohol items, the participants indicated their mean frequency of consumption in terms of the specified serving size by checking one of seven frequency categories ranging from ‘almost never’ to ‘four cups or more times/day.’ For beverage items, the participants indicated their mean frequency of consumption in terms of the specified serving size by checking one of eight frequency categories ranging from ‘almost never’ to ‘four cups or more times/day.’ Twelve dietary behaviors include 1: noodle soup meals, 2: taste of seasonings at home compared to eating out, 3: preference for the meat fat (beef and pork), 4: frequency of breakfast consumption, 5: amount of soy sauce or equivalent used for their meals, 6: frequency of soy sauce or equivalent used for meals, 7: number of side dishes at home compared to eating out, 8: amount of rice at home compared to eating out, 9: eating speed of meals, 10: intentional alterations in their eating habits using, 11: dietary supplements, and 12: receiving dietary guidance. Participants indicated a five-point scale from ‘do not like’ to ‘like very much’ for the noodle soup meals; a five-point scale from ‘light’ to ‘heavy’ for the taste of seasonings at home compared to eating out; a five-point scale from ‘do not eat’ to ‘like very much’ for the preference for the meat fat (beef and pork); a nine-point scale from ‘almost never’ to ‘daily’ for the frequency of breakfast consumption; a five-point scale from ‘very little’ to ‘very much’ for the amount of soy sauce or equivalent used for their meals; a five-point scale from ‘almost never’ to ‘always’ for the frequency of soy sauce or equivalent used for their meals; a five-point scale from ‘more at home’ to ‘more eating out’ for the number of side dishes at home compared to eating out and the amount of rice at home compared to eating out; a five-point scale from ‘very slow’ to ‘very fast’ for the eating speed of meals; a two-point scale choosing ‘positive’ or ‘negative’ for the intentional alterations in their eating habits; a seven-point scale from ‘almost never’ to ‘two or more times/day’ for the dietary supplements; and a two-point scale choosing ‘received’ or ‘not received’ for the receiving dietary guidance. For cooking methods of different fish and meats, the participants indicated the frequency of consuming raw fish on a seven-point scale ranging from ‘almost never’ to ‘two times/day.’ The same protocol was followed for boiled fish, grilled fish, tempura/fried fish, grilled meat, grilled meat pate, fried meat, stir fried meat, and stewed meat. The reproducibility and validity of the BDHQ have been described in detail in a previous study [15].

### Clustering dietary patterns using unsupervised machine learning

**Fig. 2:**
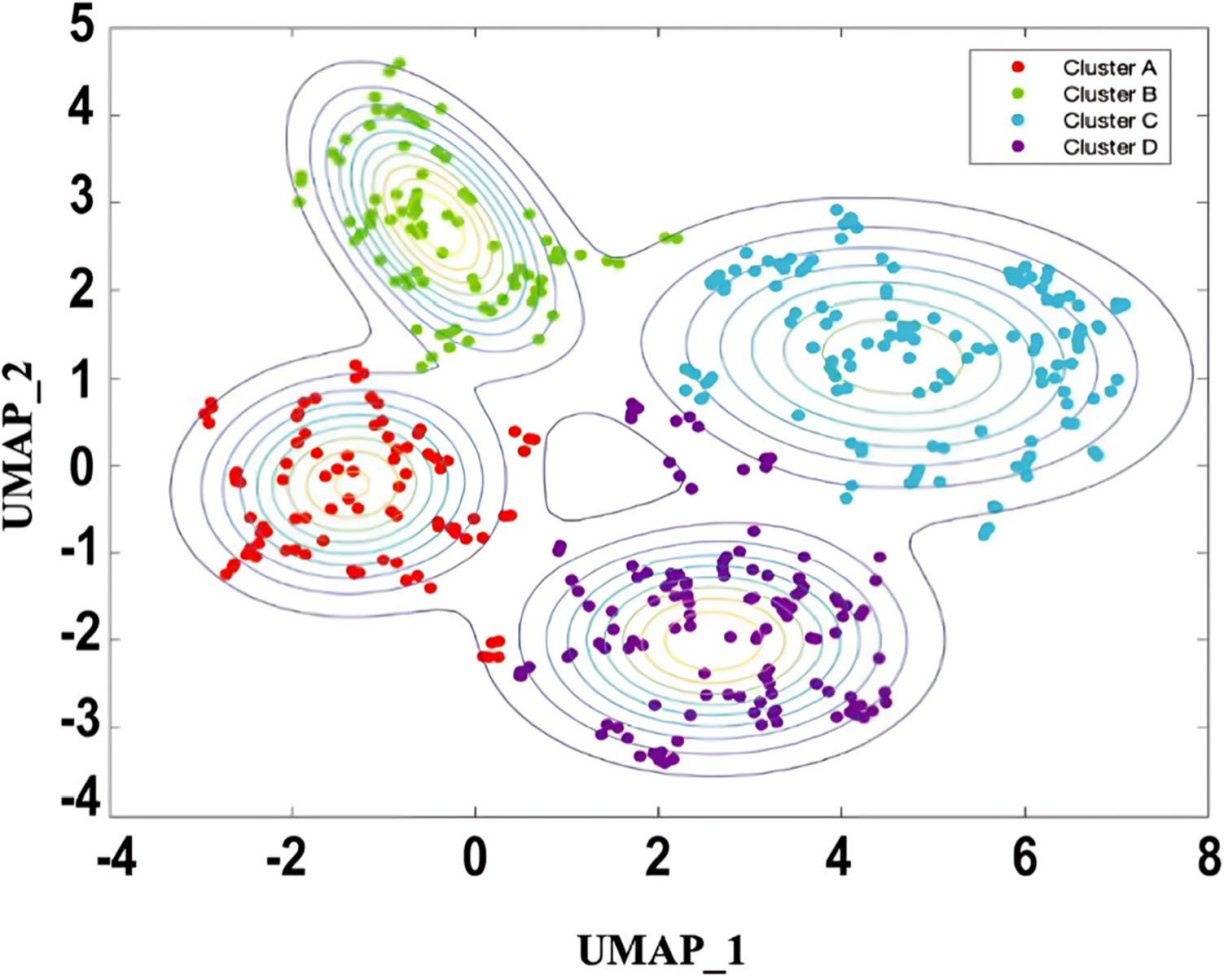
Scatter plot and contour map. The graph illustrates the relative distances between participants in the Oroshisho cohort study using unsupervised machine learning and dimension reduction techniques based on BDHQ data. The data has been reduced to two dimensions (Dimension 1 (D1) and Dimension 2 (D2)) using UMAP. Each point on the graph represents an individual participant in the study. The contour lines are curves that show areas of constant Gaussian values in the 2D space, connecting points of similar 2D values. Number clusters (*n* = 4) were chosen optimally by contour map analysis. Exemplary 2D visualization of the relative distances between all participants in the Oroshisho cohort study using UMAP. Colors indicate cluster assignment using K-means clustering (*K* = 4). An interpretable name for the dietary pattern is then defined for each cluster based on the trend value (see ‘Methods’ for details)

Analysis with unsupervised machine learning used all 79 variables, including 58 foods and non-alcoholic beverage items, 12 dietary behaviors, and 9 cooking methods common in the general Japanese population [44]. We applied dimension reduction using UMAP [38, 39] and a subsequent clustering using the K-means algorithm [45] using the raw data. UMAP dimension reduction (reduce_dimension) was performed using the following parameters: umap_min_dist = 0.1, max_components = 2, clustering neighbors (n_neighbors) = 10, and ‘Chebyshev’ as the metric. We determined the values of each parameter after the parameter tuning, in which each parameter was changed in a certain range to produce well-separated cluster distribution. 2D data were applied to K-means clustering; the default settings were used for K-means clustering. The number of clusters was determined using the elbow method and silhouette analysis [28, 46] (Supplementary Fig. 2) and the final cluster number was also confirmed as peak numbers in the contour map (Fig. 2).

To quantitatively evaluate the dietary patterns among the clusters, we introduced a “trend score” converted from the original BDHQ scale. First, the BDHQ scale was transformed into trend values as “weight” shown in Table 1. Because the proportion of each “weight” (Supplementary Fig. 1) could be suitable for understanding patterns among valuables or clusters and not for performing quantitative comparisons, the “proportion” of each “weight” against the total number was calculated (Supplementary Fig. 1). Finally, the “weight” was multiplied by the “proportion” and summed up to obtain the “trend score” for each variable.

$${\text{Trend score }} = { }\mathop \sum \limits_{i = 1}^{n} \varpi i \cdot \pi i,$$

where *ω* and *π* are “weight” and “proportion”, respectively.

**Table 1:** Components and point value for scoring the trend scores

Food groups	Food group components	Trend values
Meat	Poultry, meat, processed meat, liver	7 points for “2 or more times per day,”6 points for “1 time per day,” 5 points for “4–6 times per week,” 4 points for “2–3 times per week,”3 points for “1 time per week,” 2 points for “less than 1 time per week,” 1 point for “never”;
Fish	Squid and octopus, shellfish, fish with bone, tuna, oily fish, dried fish, lean fish, fish paste	
Vegetables	Raw vegetables used in salad, green leafy vegetables, broccoli, cabbage, Chinese cabbage, carrots, pumpkins, radishes and turnips, other root vegetables (onions, burdock, lotus root), tomato sauce, boiled tomato, and stewed tomato, salted vegetable pickles	
Fruits	Citrus fruits, strawberries, persimmons, kiwi fruit, other fruits	
Cereals	Bread: including white bread and sweet Japanese bread Noodles: buckwheat noodles, Japanese wheat noodles; instant noodles, Chinese noodles, spaghetti, macaroni	
Seasonings/condiments/cooking	Butter, margarine, jams, mayonnaise, ketchup, soy, and other sauces	
Confectionaries	Cookies, biscuits, Japanese sweets, rice crackers, rice cakes, Japanese-style pancakes, snack confectionaries	
Beverages	Green tea, black tea, oolong tea, coffee, fruits and vegetable juice, sugar-sweetened beverages (cola, sweetened soft drink, coffee with milk, lactobacillus beverage)	Eight points for “four cups or more per day,” seven points for “two to three cups per day,” six points for “one cup per day,” five points for “four to six cups per week,” four points for “two to three cups per week,” three points for “pne cup per week,” two points for “less than one cup per week,” one point for “never”
Dairy products	Milk, low-fat milk, yogurt, cheese	Seven points for “two or more times per day,” six points for “one time per day,” five points for “four to six times per week,” four points for “two to three times per week,”three points for “one time per week,” two points for “less than one time per week,” one point for “never”
Fermented soybean paste, breakfast, rice, and alcohol (unspecified)	Miso, breakfast, rice, and alcohol frequency	Nine points for “one time per day,” eight points for “six times per week,” seven points for “five times per week,” six points for “four times per week,” five points for “three times per week,” four points for “ two times per week,” three points for “ one time per week,” two points for “ less than one time per week,” one point for “never”
Alcohol (specified type)	Nihonshu/Japanese sake (rice wine), shochu (distilled spirits), beer, whisky, wine	Seven points for “4 cups or more per day,” six points for “3 cups per day,” five points for “2 cups per day,” four points for “1 cup per week,” three points for “0.5 cup per week,” two points for “less than 0.5 cup per week,” one point for “never”;
Dietary behavior		The general dietary behavior was converted into scores of 5, 4, 3, 2, 1, respectively. Using the example of soy sauce usage frequency: five points for "Always use," four points for "Often use," three points for "Sometimes use," two points for "Rarely use," one point for "Never use"

Unsupervised machine learning algorithms were executed using Python programming language (version 3.7.10) deployed by Anaconda (version 2.1.1) in the Mac operating system (version 12.5). In addition, all necessary libraries (umap-learn version 0.3.10, scikit-learn version 0.21.3) were installed on a Jupyter Notebook (version 6.4.6).

### Dietary approach to stop hypertension (DASH) score

A 40-point DASH score was estimated according to the previous study [47] to evaluate our dataset conformity to a DASH-style diet. The score comprises 8 dietary components, with recommended increases in the consumption of certain items (fruits, vegetables, nuts and legumes, low-fat dairy, and whole grains) and restrictions on others (sodium, sweetened beverages, and red and processed meats). Participants' sub-scores (1 to 5) for each component were determined based on sex-specific quintiles. The final DASH score, ranging from 8 to 40, was derived by summing the sub-scores for each component.

### Absolute intake and relative intake

Absolute intake of each food item was obtained with BDHQBOX service (EBNJAPAN, DHQ Support Center, Tokyo, Japan). Absolute intake estimated from the BDHQ is based on Japan's Standard Tables of Food Composition (5th edition) [56]. Relative intake was calculated using the equation below:

$${\text{Relative intake }} = \, \left( {{\text{absolute intake}}/{\text{total energy intake}}} \right),$$

where total energy intake was also obtained with BDHQBOX service.

### Salt intake estimation

The estimated values of daily salt intake were also obtained with the BDHQBOX service (EBNJAPAN, DHQ Support Center, Tokyo, Japan) according to the previous studies [99, 100].”

### Grouping food items

By consolidating similar food items from the original intake data, we generated a unified representation, yielding a new dataset that captures aggregated dietary patterns by PCA. The process entailed combining categories, such as fish, milk, beverages, eggs, noodles, desserts, meats, and vegetables, into composite data using average intake frequencies. A new dataset was presented in Supplementary Data 1.

### Assessment of covariates

Other lifestyle-related and sociodemographic information, including age, smoking status, sleep duration, living status (alone), and educational level (≥ college or not), were obtained using a self-administered questionnaire. The average sleep duration per night was estimated based on when the participants went to bed and awoke without considering of week or weekend and categorized into ‘6–8 h per day’ (typical sleep duration) or not (atypically shorter or longer). Anthropometric parameters (height, body weight, and waist circumference) were measured according to a standardized protocol. The body mass index (BMI) was calculated as weight/height^2^ (kg/m^2^). Educational level was assessed by determining the final grade level and was divided into two categories: “less than college” and “college or above”.

Physical activity (PA) was measured using the International Physical Activity Questionnaire (IPAQ) [48]. Total weekly PA (metabolic equivalents [METs] × hours/week) was calculated and classified into three categories: 0 (The '0' category represents individuals in a state of rest or very low activity at baseline [42, 48]), 0.1–22.9, and ≥ 23 (METs × hours/week), the recommended level of physical activity for preventing lifestyle-related diseases in Japan is represented by the category ≥ 23 METs × hours/week [42, 48]. Depressive symptoms were assessed using the Japanese version of the Self-Rating Depression Scale (SDS) [49]. The SDS score ranging from 0 to 100, was used to evaluate the severity of depressive symptoms. An SDS score ≥ 40 was the cutoff point for relatively mild or severe depressive symptoms [50]. A score below 40 indicates the absence of significant depressive symptoms, while a score of 40 or higher suggests mild to severe depressive symptoms [50]. Blood samples were collected in siliconized vacuum glass tubes containing sodium fluoride to analyze the levels of fasting blood glucose, uric acid (UA), and lipids. Fasting blood glucose (FBG) was measured using an enzymatic method (Eerotec, Tokyo, Japan). Serum UA levels were measured enzymatically using a Pureauto SUA kit (Sekisui Medical Co. Ltd., Tokyo, Japan). The concentrations of triglycerides, low-density lipoprotein cholesterol (LDL-C), and high-density lipoprotein cholesterol (HDL-C) were measured by enzymatic methods using the appropriate kits. Moreover, a history of physical illness and current medication, including diabetes and dyslipidemia, were noted from ‘yes’ or ‘no.’ Diabetes was defined as an FBG level > 126 mg/dL, or a self-reported history of previously diagnosed diabetes or current medication for diabetes was regarded as having diabetes [51, 52]. Dyslipidemia was defined as a low-density lipoprotein cholesterol level ≥ 140 mg/dL, a high-density lipoprotein cholesterol level < 40 mg/dL, a triglyceride level ≥ 150 mg/dL, or the use of medications for dyslipidemia [53].

### Statistical analysis

JMP software (version 16.2.0) was used for statistical analyses. The normality of variables was checked using the Kolmogorov–Smirnov test [54]. Continuous variables were checked for normality and transformation was not required. Descriptive statistics (i.e., mean and standard deviation [SD] for continuous variables and frequencies and percentages for categorical variables) were reported for all variables by cluster. ANOVA for continuous variables and Chi-square tests for categorical variables were used to assess cluster differences. Proportional band graphs were used to evaluate cluster features for each variable (Supplementary Material 1).

Multivariable logistic regression analysis was used to examine the association between each cluster and the incidence of hypertension. Hypertension was used as the dependent variable and the cluster of dietary patterns was used as the independent variable. The odds ratios (ORs) and 95% confidence intervals (CIs) were calculated using the likelihood ratio method to test, the p value for the incidence of hypertension compared to a reference cluster. Model 1 was a crude model. Model 2 was adjusted for age and BMI at the baseline. For model 3, all variables in model 2 and baseline smoking status, PA, and education were used. Model 4 was further adjusted for baseline dyslipidemia and diabetes mellitus. Model 5 was further adjusted for salt intake, a potential mediator, to examine the extent to which the association between different dietary patterns and hypertension could be attenuated.

Sensitivity analysis was used to examine the impact of residual confounding of age on the association between clusters characterized by dietary patterns and the incidence of hypertension. Furthermore, two analyses were performed; first, we checked the age distribution across the dietary pattern groups. We used box plot diagrams to determine whether there was sufficient age overlap among men of different cluster statuses to examine the effects of clusters and age separately. Next, we performed an age-matched group analysis. Each participant in each group was age-matched based on two matched ranges: ± 0 and ± 2 years. For each participant in cluster A, participants are selected from clusters B, C, and D who fall within the specified age ranges. These age ranges are ± 0 years (meaning participants with exactly the same age) and ± 2 years (participants who are within a 2-year age range of the participant from cluster A). We then performed the same multivariable logistic regression analysis as the main analysis. The same analysis methods as for the primary outcome were used to compare the use of food items alone with the outcome of combing food items, cooking methods, and dietary behavior to be driven dietary patterns with the incidence of hypertension. A *P* value < 0.05 was considered statistically significant in all analyses.

## Results

### Dietary patterns clustered using unsupervised machine learning

A dataset with 79 dimensions (79 variables) was reduced to two dimensions using the UMAP algorithm. The first and second dimensions were plotted on the *x* and *y* axes. A suitable number of clusters was determined to be four using the elbow method (see Supplementary Fig. 2). A 2D scatter plot of the clusters is presented in Fig. 2. The 2D scatter plot was overlaid with a density contour map showing four peaks consistent with the cluster number (Fig. 2).

### Characteristics of dietary patterns by the trend scores

Instead of using the original BDHQ sore, we introduced a “trend score” (Table 2) to compare quantitatively the dietary patterns among the clusters (see “Methods” for details). The characteristics description for each cluster is below. The statistical significance was also examined among clusters for each variable. Cluster A was characterized by the highest consumption of western-type confectioneries (2.880), pasta (2.453), coffee (5.987), cola drink/soft drink (3.880), sugar (1.880), and persimmon-seasonal (3.347) in the food items; noodle soup (3.560) and meat fat (beef and pork) (3.093) in the dietary behaviors. Cluster A showed the lowest scores in many food items (26/58 items), especially proteins and vegetables, and the cooking methods (7/9 methods). The participants in this cluster tended to skip breakfast and appear to have unhealthy dietary behaviors such as taking noodle soup (high salt intake) or meat fat (high fat intake). The identified pattern was named the ‘Low-protein/fiber High-sugar’ pattern. Cluster B was characterized by the highest scores in many food items (29/58 items), especially dairy products, vegetables, and fruits, the cooking methods (5/9 methods), and dietary behaviors (6/12 behaviors). This cluster appeared to show healthy dietary behaviors and was named the ‘Dairy/vegetable-based’ pattern. Cluster C was characterized by the highest scores of chicken meat (3.190) and pork/beef (3.720), mayonnaise (3.700), bread (3.980), Chinese noodles (3.140), and beer (3.530) in the food items; grilled meat/steak (2.530) in the cooking methods; frequency of soy sauce or equivalent used for meals (3.790), amount of soy sauce or equivalent used for meals (3.020), and supplements (1.850) in the dietary behaviors. The Cluster C was referred to as the ‘Meat-based’ pattern. Cluster D was characterized by the highest consumption of most seafood (5/6 items) and alcohol (5/6 items) in the food items and fish-related items in the cooking methods (4 methods). This cluster was named the ‘Seafood and Alcohol’ pattern.

**Table 2:** Dietary patterns identified by the trend score

Variables	Cluster A ‘Low-Fiber High-Sugar’	Cluster B ‘Dairy/vegetable-based’	Cluster C ‘Meat-based healthy’	Cluster D ‘Seafood and Alcohol’	*P* value
Food items					
Reduced fat milk and yogurt	2.053	2.769	2.270	**2.535**	0.5909
Milk and yogurt	**2.933**	3.139	2.520	2.768	0.4589
Chicken meat	3.093	3.046	**3.190**	3.092	0.6273
Pork/beef	3.640	3.608	**3.720**	3.528	0.9317
Ham/sausage/bacon	3.107	**3.277**	3.240	3.239	0.3610
Liver	1.347	1.354	1.390	**1.416**	0.2421
Squid/octopus/shrimp/shellfish	2.480	2.615	2.670	**2.782**	0.3688
Small fish with bones	1.840	1.923	1.950	**2.204**	0.1726
Cannes tuna	1.720	**1.831**	1.770	1.655	0.7504
Dried fish /salted fish	2.253	2.531	2.480	**2.761**	0.3743
Oily fish	2.427	2.662	2.500	**2.761**	0.2513
Lean fish	2.387	2.723	2.640	**2.901**	**0.0170**
Egg	3.587	4.185	3.710	**4.232**	**0.0034**
Tofu/fried tofu	3.480	**3.962**	3.600	3.887	**0.0188**
Natto	2.800	3.585	2.930	**3.655**	**0.0008**
Potatoes	2.933	**3.631**	2.990	3.366	**0.0013**
Pickled green leafy vegetables	3.413	**3.785**	3.380	3.732	0.0986
Other pickled vegetables	2.947	3.439	3.480	**3.697**	**0.0068**
Lettuce/cabbage (raw)	3.920	**4.246**	3.840	4.070	0.2920
Green leafy vegetables	3.267	3.446	3.190	**3.613**	0.2236
Cabbage/Chinese cabbage	3.347	3.723	3.460	**3.747**	0.1424
Carrots/pumpkin	3.080	**3.500**	3.160	3.317	0.0933
Japanese radish/turnip	2.867	**3.315**	2.880	3.211	0.1127
Other root vegetables	3.240	**3.677**	3.410	3.648	0.3931
Tomatoes	3.067	**3.400**	3.060	3.239	0.6047
Mushrooms	2.880	**3.069**	2.920	3.070	0.9113
Seaweeds	3.013	**3.392**	3.180	3.345	0.3371
Western-type confectioneries	**2.880**	2.731	2.530	2.366	**0.0399**
Japanese-type confectioneries	2.133	**2.292**	1.960	2.000	0.4327
Senbei/rice crackers/rice cake/okonomiyaki	2.573	**2.977**	2.480	2.528	**0.0030**
Ice cream	2.800	**3.015**	2.510	2.106	** < .0001**
Citrus fruit	2.093	**2.231**	1.810	2.077	0.5068
Persimmons/strawberries/kiwifruit	1.760	**1.885**	1.530	1.648	0.1610
Other fruits (e.g., apples and bananas etc.)	2.987	**3.408**	2.720	2.880	0.1753
Mayonnaise	3.547	3.562	**3.700**	3.599	0.5772
Bread	3.893	3.839	**3.980**	3.261	**0.0026**
Buckwheat noodles	2.787	**2.839**	2.830	2.740	0.7921
Japanese wheat noodles	2.547	**2.839**	2.730	2.676	0.5504
Chinese noodles	3.013	2.862	**3.140**	2.775	0.0665
Pasta	**2.453**	2.362	2.230	2.014	**0.0159**
Green tea	5.080	**5.446**	4.970	5.077	0.2474
Black tea/oolong tea	3.507	**3.800**	3.370	3.049	0.4669
Coffee	**5.987**	5.185	5.820	5.007	**0.0033**
Cola drink/soft drink	**3.880**	3.777	3.340	3.225	0.4197
100% juice and vegetable juice	2.973	**3.023**	2.560	2.387	0.1040
Other grains (e.g., barley, white rice with germ, brown rice, and whole grains, etc.)	1.453	**1.539**	1.390	1.486	0.4702
Sugar	**1.880**	1.654	1.630	1.549	0.0802
Rice	4.187	**4.554**	4.050	4.282	**0.0033**
Miso soup	3.240	3.485	3.030	**3.542**	0.0584
Alcohol frequency	2.067	3.039	6.820	**8.064**	** < .0001**
Nihonshu/Japanese sake (rice wine)	1.320	1.354	1.600	**1.796**	0.2726
Beer	2.267	2.646	**3.530**	3.120	** < .0001**
Shochu (distilled spirits)	1.893	2.123	3.280	**3.479**	** < .0001**
Whisky	1.147	1.254	1.280	**1.430**	0.7963
Wine	1.067	1.300	1.130	**1.380**	0.1949
Citrus fruits—seasonal	3.533	**3.669**	3.040	3.268	0.1169
Persimmon—seasonal	**3.347**	2.377	1.960	2.345	**0.0203**
Strawberry—seasonal	2.560	**2.846**	2.420	2.704	0.6147
Cooking methods					
Raw fish (Sashimi/sushi)	2.560	2.831	2.910	**3.085**	**0.0030**
Grilled fish	2.987	3.323	2.990	**3.437**	**0.0427**
Boiled fish/fish served in pot/fish in soup	2.933	3.277	3.050	**3.394**	0.2337
Tempura/fried fish	2.320	**2.492**	2.360	2.366	0.7177
Grilled meat/steak	2.453	2.362	**2.530**	2.345	0.3302
Hamburg steak/curry/meat sauce	2.707	**2.739**	2.720	2.486	0.0975
Deep fried meat/Tempura meat	2.680	**2.731**	2.700	2.704	0.9645
Stir fried meat	3.187	**3.446**	3.410	3.366	0.0978
Stewed meat/boiled meat/meat served in pot or bowl/meat in soup	3.600	**4.185**	3.670	4.077	**0.0465**
Dietary behaviors					
Noodle soup	**3.560**	3.369	3.400	3.458	0.9282
Taste of seasonings at home vs. Eating out	3.387	3.508	3.310	**3.528**	0.0474
Meat fat (beef and pork)	**3.093**	2.792	2.810	2.916	0.1280
Breakfast	2.227	**8.600**	2.580	8.451	** < .0001**
Frequency of soy sauce or equivalent used for meals	3.600	3.715	**3.790**	3.711	0.3384
Amount of soy sauce or equivalent used for meals	3.000	2.908	**3.020**	2.845	0.2721
Number of side dishes at home compared to eating out	3.107	**3.246**	3.090	3.232	0.2309
Amount of rice at home compared to eating out	3.013	**3.016**	2.940	2.789	0.4306
Speed of eating	3.520	**3.531**	3.490	3.303	0.6580
Conscious changes in eating habits	1.387	**1.654**	1.440	1.423	0.1169
Supplements	1.400	1.785	**1.850**	1.753	0.5737
Dietary guidance	1.027	**1.039**	1.030	1.021	0.8633

*P* values less than 0.05 are indicated as bold

### Characteristics according to cluster categories of dietary patterns

Table 3 presents the characteristics of the clusters of dietary patterns. Differences were observed in lifestyle parameters; we observed heterogeneity in these variables across all the patterns. The mean age and BMI were higher in the ‘Dairy/vegetable-based’ pattern (age: 44.88 ± 10.5 years, BMI: 23.73 ± 3.14) and the ‘Seafood and Alcohol’ pattern (age: 47.58 ± 9.69 years, BMI: 23.08 ± 2.84) compared to ‘Low-Fiber High-Sugar’ pattern (age: 40.25 ± 8.27 years, BMI: 22.90 ± 2.48) and ‘Meat-based’ pattern (age: 43.74 ± 8.86 years, BMI: 22.75 ± 2.69). The ‘Meat-based’ pattern exhibited a higher proportion of eating dinner before bedtime (67.0%), smoking (70%), or skipping breakfast (64.0%). However, the distribution of education level and sleep quality was different across all patterns (*P* < 0.05). Additionally, differences were observed in diagnostic and blood examination parameters; participants in the ‘Low-Fiber High-Sugar’ pattern exhibited the lowest level of SBP (118.05 ± 11.57 mmHg), DBP (72.16 ± 8.90 mmHg), and fasting glucose (91.29 ± 8.74), and demonstrated the highest level of LDL-C (128.24 ± 32.60 mg/dL). Compared with other patterns, the ‘Seafood and Alcohol’ pattern had a higher proportion of diabetes (9.9%) and the highest level of HDL-C (56.65 ± 13.19 mg/dL). Table 3 also shows that the participants in the 'Dairy/vegetable-based' pattern (12.05 ± 4.15) and 'Seafood and Alcohol' pattern (11.56 ± 3.06) were taking higher amount of salt than those in the 'Low-Fiber High-Sugar' pattern (10.45 ± 2.55) and 'Meat-based' pattern (10.93 ± 2.85). No association was observed between the clusters and the other factors.

**Table 3:** Demographic characteristics of the four clusters

Variables	Cluster A ‘Low-Fiber High-Sugar’	Cluster B ‘Dairy/vegetable-based’	Cluster C ‘Meat-based healthy’	Cluster D ‘Seafood and Alcohol’	*P* value
No. of participants	75	130	100	142	
Hypertension, % (*n*)	8.00 (6)	13.08 (17)	9.00 (9)	22.53 (32)	
Age (years)	40.25 ± 8.27	44.88 ± 10.50	43.74 ± 8.86	47.58 ± 9.69	< .001
BMI (kg/m^2^)	22.90 ± 2.48	23.73 ± 3.14	22.75 ± 2.69	23.08 ± 2.84	0.046
Waist circumference, cm	81.33 ± 7.26	83.83 ± 8.82	81.90 ± 7.84	83.34 ± 8.18	0.098
Snacking after dinner, % (*n*)	20.0 (15)	16.9 (22)	15.0 (15)	8.5 (12)	0.082
Eating dinner before bedtime, % (*n*)	53.3 (40)	38.5 (50)	67.0 (67)	60.6 (86)	< .001
Skipping breakfast, % (*n*)	58.7 (44)	6.9 (9)	64.0 (64)	5.6 (8)	< .001
Living status (alone), % (*n*)	36.0 (27)	25.4 (33)	26.0 (26)	12.7 (18)	< .001
Sleep duration (≥ 6 and ≤ 8 h/day), % (*n*)	48.0 (36)	53.1(69)	55.0 (55)	61.3 (87)	0.269
Education (college or more), % (*n*)	28.0 (21)	43.8 (57)	36.0 (36)	27.5 (39)	0.022
Sleep quality (poor), % (*n*)	49.3 (37)	37.7 (49)	54.0 (54)	33.1 (47)	0.004
Smoking status, % (*n*)					< .001
Never smoker	33.3 (25)	48.5 (63)	20.0 (20)	33.8 (48)	
Former smoker	8.0 (6)	11.5 (15)	10.0 (10)	14.8 (21)	
Current smoker	58.7 (44)	40.0 (52)	70.0 (70)	51.4 (73)	
Physical activity, % (*n*)					0.157
0 METs h/week	20.0 (15)	22.3 (29)	29.0 (29)	27.5 (39)	
0.1–22.9 METs h/week	41.3 (31)	36.2 (47)	43.0 (43)	32.4 (46)	
≥ 23 METs hour/week	38.7 (29)	41.5 (54)	28.0 (28)	40.1 (57)	
Depressive symptoms, % (*n*)					0.943
No	86.7 (65)	88.5 (115)	88.0 (88)	89.4 (127)	
Yes	13.3 (10)	11.5 (15)	12.0 (12)	10.6 (15)	
Dyslipidemia, % (*n*)					0.335
No	48.0 (36)	50.8 (66)	58.0 (58)	58.5 (83)	
Yes	52.0 (39)	49.2 (64)	42.0 (42)	41.5 (59)	
Diabetes, % (*n*)					< .001
No	100 (75)	98.5 (128)	99.0 (99)	90.1 (128)	
Yes	0.0 (0)	1.5 (2)	1.0 (1)	9.9 (14)	
SBP, mmHg	118.05 ± 11.57	121.80 ± 11.41	119.80 ± 9.53	124.69 ± 10.02	< .001
DBP, mmHg	72.16 ± 8.90	75.40 ± 8.79	74.22 ± 7.76	77.47 ± 7.26	< .001
Cholesterol, mg/dL	195.52 ± 32.80	198.83 ± 30.78	194.39 ± 33.72	192.01 ± 28.03	0.422
Triglycerides, mg/dL	116.01 ± 59.75	125.03 ± 74.25	126.91 ± 86.30	137.98 ± 96.43	0.281
LDL-C, mg/dL	128.24 ± 32.60	125.39 ± 28.47	120.18 ± 33.92	111.66 ± 25.41	0.001
HDL-C, mg/dL	50.70 ± 10.96	50.66 ± 13.19	55.06 ± 15.67	56.65 ± 13.19	0.005
Glucose, mg/dL	91.29 ± 8.74	92.83 ± 12.93	92.38 ± 10.98	97.92 ± 21.89	0.005
Uric acid, mg/dL	5.91 ± 1.13	6.05 ± 1.13	5.99 ± 1.08	6.07 ± 1.22	0.824
Hemoglobin, g/dL	14.66 ± 0.95	14.69 ± 0.91	14.72 ± 0.94	14.72 ± 0.89	0.968
Salt intake, (g/day)	10.45 ± 2.55	12.05 ± 4.15	10.93 ± 2.85	11.56 ± 3.06	0.004

This table presents the demographics, blood tests, and clinical characteristics of the four clusters. Group comparisons were conducted using Chi-square tests or analysis of variance (mean ± SD in parentheses). *P* values are from one-way analysis of variance (ANOVA) or Chi-square tests as appropriate. *BMI* body mass index, *SBP* systolic blood pressure, *DBP* diastolic blood pressure, *FBG* fasting blood glucose, *RBC* red blood cells, *HB* hemoglobin, *WBC* white blood cells, *PA* physical activity, *LDL-C* low-density lipoprotein cholesterol, *HDL-C* high-density lipoprotein cholesterol

### Association of clusters with hypertension

Table 4 presents the ORs and 95% CI for the incidence of hypertension according to the clusters of dietary patterns. We attempted to identify a dietary pattern with a high risk of hypertension and chose cluster D as a reference to reduce multiple comparisons since the cluster showed the largest sample size and the highest hypertension prevalence, indicating it could be an aversive dietary pattern. As indicated in the crude model with the ‘Seafood and Alcohol’ dietary pattern as the reference, the mean OR for hypertension according to clusters was 0.29 (95% CI 0.12–0.75) for the ‘Low-Fiber High-Sugar’ pattern, 0.517 (95% CI 0.272–0.984) for ‘Dairy/vegetable-based’ pattern, and 0.339 (95% CI 0.154–0.749) for ‘Meat-based’ pattern (*P* = 0.006). Even after adjustment for age, and BMI (Model 2), a similar association was still maintained (*P* = 0.049). Although the association was attenuated (*P* = 0.055) after adjusting for age, BMI, smoking, education, and PA (Model 3), the difference was confirmed after a fully adjusted model 4 (*P* = 0.014). The difference in the mean OR for hypertension between the ‘Low-Fiber High-Sugar,’ ‘Dairy/vegetable-based,’ and ‘Meat-based’ dietary patterns was confirmed in the fully adjusted model 4, with respective ORs of 0.34 (95% CI 0.13–0.91), 0.39 (95% CI 0.19–0.80), and 0.37 (95% CI 0.16–0.86). In Model 5, the same association remained (*P* = 0.018) even after adjusting for salt intake, with respective ORs of 0.35 (95% CI 0.13, 0.92), 0.43 (95% CI 0.21, 0.86), and 0.38 (95% CI 0.16, 0.86).

**Table 4:** Odds ratios for the incident hypertension by cluster

	Cluster D ‘Seafood and Alcohol’	Cluster A ‘Low-Fiber High-Sugar’	Cluster B ‘Dairy/vegetable-based’	Cluster C ‘Meat-based healthy’	*P* value
Model 1	1.00 (ref)	0.29 (0.12, 0.75)*	0.52 (0.27, 0.98)*	0.33 (0.15, 0.75)*	0.006
Model 2	1.00 (ref)	0.40 (0.15, 1.06)	0.51 (0.26, 0.99)*	0.40 (0.18, 0.91)*	0.049
Model 3	1.00 (ref)	0.41 (0.16, 1.09)	0.47 (0.24, 0.94)	0.43 (0.19, 0.98)	0.055
Model 4	1.00 (ref)	0.34 (0.13, 0.91)*	0.39 (0.19, 0.80)*	0.37 (0.16, 0.86)*	0.014
Model 5	1.00 (ref)	0.35 (0.13, 0.92)*	0.43 (0.21, 0.86)*	0.38 (0.16, 0.86)*	0.018

Model 1: cluster. Model 2: cluster, age, BMI. Model 3: cluster, age, BMI, smoking, education, PA. Model 4: cluster, age, BMI, smoking, education, PA, dyslipidemia, diabetes. Model 5: cluster, age, BMI, smoking, education, PA, dyslipidemia, diabetes, salt intake. Significantly different from cluster D, *P < 0.05

### Age-matched analysis (sensitivity analysis)

**Fig. 3:**
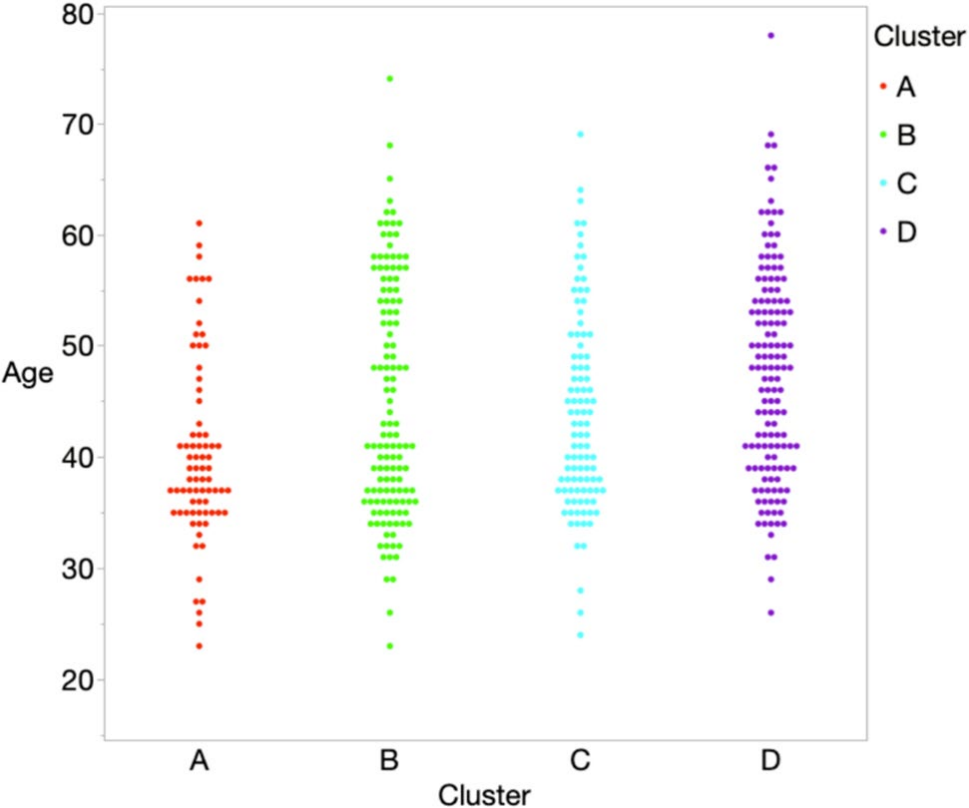
Analysis of age distribution of clusters. Dot plot of the age distribution for each cluster. The color numbering indicates a representation of each cluster

As ca be seen in Fig. 3, the distribution of age was imbalanced in each cluster. When we performed an age-matching group analysis, similar associations were obtained in the age-matched analyses (Tables 5, 6). In the analyses comparing age-matched groups, an association was observed with a range of ± 0 and ± 2 years old, respectively, with p-values of 0.030 and 0.009. Furthermore, the same association remained significant (*P* = 0.077, *P* = 0.012) even after adjusting for salt intake. However, the age-matched analysis with the range of ± 2 years old did not detect the difference between the “Low-Fiber High-Sugar” pattern and the “Seafood and Alcohol” pattern (Table 6).

**Table 5:** Odds ratios for incident hypertension by cluster (age matched ± 0)

	Cluster D ‘Seafood and Alcohol’	Cluster A ‘Low-Fiber High-Sugar’	Cluster B ‘Dairy/vegetable-based’	Cluster C ‘Meat-based healthy’	*P* value
Model 1	1.00 (ref)	0.28 (0.10, 0.73)*	0.32 (0.12, 0.83)*	0.38 (0.14, 0.94)*	0.030
Model 2	1.00 (ref)	0.27 (0.10, 0.75)*	0.30 (0.11, 0.81)*	0.37 (0.14, 0.95)*	0.025
Model 3	1.00 (ref)	0.29 (0.11, 0.81)*	0.32 (0.12, 0.86)*	0.40 (0.15, 1.05)	0.044
Model 4	1.00 (ref)	0.31 (0.11, 0.87)*	0.34 (0.12, 0.94)*	0.43 (0.16, 1.12)	0.073
Model 5	1.00 (ref)	0.32 (0.11, 0.89)*	0.34 (0.12, 0.93)*	0.44 (0.17, 1.17)	0.077

Model 1: cluster. Model 2: cluster, BMI. Model 3: cluster, BMI, smoking, education, PA. Model 4: cluster, BMI, smoking, education, PA, dyslipidemia, diabetes. Model 5: cluster, BMI, smoking, education, PA, dyslipidemia, diabetes, salt intake. Significantly different from cluster D, *P < 0.05

**Table 6:** Odds ratios for incident hypertension by cluster (age matched ± 2)

	Cluster D ‘Seafood and Alcohol’	Cluster A ‘Low-Fiber High-Sugar’	Cluster B ‘Dairy/vegetable-based’	Cluster C ‘Meat-based healthy’	*P* value
Model 1	1.00 (ref)	0.48 (0.22, 1.08)	0.35 (0.15, 0.82)*	0.35 (0.15, 0.82)*	0.034
Model 2	1.00 (ref)	0.54 (0.23, 1.23)	0.28 (0.12, 0.69)*	0.39 (0.16, 0.95)*	0.026
Model 3	1.00 (ref)	0.57 (0.24, 1.35)	0.25 (0.09, 0.63)*	0.37 (0.15, 0.94)*	0.015
Model 4	1.00 (ref)	0.52 (0.22, 1.25)	0.23 (0.09, 0.58)*	0.34 (0.13, 0.86)*	0.009
Model 5	1.00 (ref)	0.53 (0.23, 1.26)	0.24 (0.09, 0.61)*	0.34 (0.14, 0.87)*	0.012

Model 1: cluster. Model 2: cluster, age, BMI. Model 3: cluster, age, BMI, smoking, education, PA. Model 4: cluster, age, BMI, smoking, education, PA, dyslipidemia, diabetes. Model 5: cluster, age, BMI, smoking, education, PA, dyslipidemia, diabetes, salt intake. Significantly different from cluster D, *P < 0.05

### Absolute intake and relative intake

We also evaluate the clustering using absolute or relative intakes, instead of frequency of food item intake. However, neither cluster showed four obvious clusters (Supplementary Figs. 7 and 8). Three ambiguous or seven sparse clusters were observed in absolute or relative intakes, respectively. The number of clusters was chosen by the elbow method (Supplementary Figs. 7 and 8). The results suggest a lack of clear clustering patterns in absolute and relative intakes, indicating a distinct difference in structure or grouping. These findings highlight the complexity and variability of the data, underscoring the importance of the chosen parameter for the clustering analysis.

### Comparison to analysis using DASH scores

Regarding hypertension, the diet based on Dietary Approaches to Stop Hypertension (DASH) has been shown to reduce blood pressure in the previous studies [55, 56]. We estimated the DASH score using our dataset according to the procedure reported previously [55] (see ‘Methods’) and compared it with our result. Compared with previous studies, our data showed relatively lower DASH scores (DASH scores: 5–9 in Q1, 9–12 in Q2, 12–14 in Q3, and 14–23 in Q4) and no association with the incidence of hypertension between the lowest DASH score group and the others (Supplementary Table 1). Supplementary Table 2 shows the proportions of DASH scores ranging from Q1 to Q4 for each cluster. Cluster D, which exhibited the highest risk for hypertension and the lowest DASH score, demonstrated statistical differences in quantile composition from clusters A and B, but not cluster C. Cluster D also showed differences in average DASH scores from clusters A and B, but not cluster C (Supplementary Table 2).

### Examining parameter categories in clustering

We also examined the contribution of the category of parameters to clustering (Supplementary Table 3). When removing “cooking methods” category from the original parameter set (food items + dietary behaviors + cooking methods), four clusters were also observed (Supplementary Fig. 5) and the most fraction of clusters coincided with the clustering (overlap percentages for Clusters A = 90.7%, B = 91.5%, C = 79.0%, D = 88.7%). However, removing “dietary behaviors” or “dietary behaviors + cooking methods (only food items)” resulted in no obvious four clusters observed (Supplementary Figs. 3 and 4). Two dense clusters were observed by clustering using only “dietary behaviors” (Supplementary Fig. 5). This result suggests a critical contribution of “dietary behaviors” to clustering and dietary patterns represented as food items could become clearer by combining different categories of parameters.

### Evaluation and optimization of food cluster grouping using principal component analysis

Procedure grouping similar food items is frequently used to reduce valuables by prior knowledge or after principal component analysis. We assessed the effect of grouping parameters on our clustering approach. We reclassified similar food items into each typical category, such as yogurt and milk, into the dairy product category. The grouping results are shown in Supplementary Data 1. The grouped dataset failed to produce clear clusters (Supplementary Fig. 6).

## Discussion

This study applied unsupervised machine learning to identify dietary patterns based on food intake frequency, dietary behaviors, and cooking methods. We identified four dietary patterns: ‘Low-Fiber High-Sugar,’ ‘Dairy/vegetable-based,’ ‘Meat-based,’ and ‘Seafood and Alcohol’ patterns. A series of statistical analyses revealed that ‘Dairy/vegetable-based’ and ‘Meat-based’ patterns are associated with a lower risk of hypertension than ‘Seafood and Alcohol’ patterns. Furthermore, the results remained similar even when age-matched group analyses were performed.

Since there is no ‘gold standard’ procedure for classifying dietary patterns, pattern classification has often been based on relevant expertise or experience. Identifying dietary patterns can be applied to validated dietary assessment methods, such as food frequency questionnaires [15] or 24-h dietary recalls [57], using established dietary pattern analysis techniques, such as PCA [13] and factor analysis [15]. PCA and factor analysis, utilizing data on food intake [28, 58], can help identify the essential food groups or factors contributing to an individual’s diet and minimizes data complexity by reducing the number of variables. PCA, however, has a limitation in that it is a linear method and may not effectively capture nonlinear relationships in the data [59]. Additionally, PCA is sensitive to outliers and extreme values, which can result in distorted results [60]. Factor analysis also has limitations, including the subjective nature of defining and interpreting the factors [61], the impact of the number of factors to extract and the method of extraction from the results [61], and the assumption of a specific structure in the data, which may not always hold true [62]. Therefore, the development of new, practical dietary approaches is desirable. Combining UMAP and K-means may be a powerful tool for analyzing dietary patterns in epidemiological studies. UMAP has gained popularity for dimensionality reduction and visualization due to its capacity to reveal intricate relationships in high-dimensional data, since this method stands out for its exceptional ability to capture complex nonlinear patterns within data while preserving global structure [38]. UMAP often outperforms traditional techniques like t-SNE and PCA in terms of preserving the underlying data structure [63]. UMAP is commonly used to visualize high-dimensional datasets containing over 20,000 genes in transcriptomics or population genetics [64–66]. Recently, UMAP has begun to be applied for identifying sub-groups from demographic features or visualizing clusters of food items in epidemiological data [37, 68]. On the other hand, K-means is frequently used for its simplicity and efficiency in clustering data points, especially in scenarios where the number of clusters is known in advance, since this algorithm was shown to be highly scalable and competitive with other clustering algorithms when applied to large datasets [67]. K-means is commonly used to group similar food items and identify common dietary patterns after PCA [45, 68]. In summary, UMAP offers superior nonlinear dimensionality reduction capabilities, while K-means provides a straightforward and efficient solution for clustering tasks, as shown by our approach successfully identified dietary patterns associated with the incidence of hypertension.

Dietary patterns are not solely determined by the types of food intake but are also influenced by dietary behaviors and cooking methods [69–71]. Therefore, when evaluating dietary patterns, it is suggested that considering such dietary behavior as portion sizes, frequency of meals, speed of eating, and eating out frequency can impact the overall nutritional quality of a person's diet [72, 73]. Cooking methods also play a crucial role in determining the nutrient content, caloric intake, and fat intake of food [74, 75]. For instance, deep-frying can increase the calorie and fat content, while steaming or grilling can help retain the nutrient content of vegetables [76–78]. Considering food intake, dietary behavior, and cooking methods is crucial when evaluating dietary patterns, as this can provide a comprehensive understanding of the population's eating habits. It can also identify areas where dietary interventions can be targeted to improve overall health and well-being.

This study identified four distinct dietary patterns: Cluster A, Low-protein/fiber High-sugar; Cluster B, Dairy/vegetable-based; Cluster C, Meat-based; and Cluster D, Seafood and Alcohol, named according to the trend score ranking. Notably, 17 of 58 food items, 3 of 9 cooking methods, and 1 of 12 dietary behaviors showed differences among clusters (Table 2). However, named patterns by the ranking of the trend scores are not always consistent with differences. This could result from a complex combination of 79 variables and might reflect holistic interaction between groups or patterns of variables. However, the patterns identified demonstrated some similarities to previous studies among Japanese adults. For example, the ‘Dairy/vegetable-based’ pattern was similar to the ‘Traditional Japanese’ pattern (vegetables, fruits, potatoes, mushrooms, seafood, grains, milk, legumes, and low intake of beverages) identified by the nutritional dietary survey [16]. Additionally, the ‘Sweet-fat’ pattern (Western and Japanese confectioneries, mayonnaise and other dressings, bread, and ice cream) was previously identified similarly in the Nagano Nutrition and Health Study [79]. The ‘Fast Food/Sweet’ pattern (ice cream, desserts, chocolate, soft drink, and bread) from Qatar Biobank survey was similar to the ‘Low-Fiber High-Sugar’ patterns identified in this study [80].

Furthermore, the ‘Seafood and Alcohol’ pattern was similar to the ‘Izakaya’ or ‘Seafood-Alcohol’ patterns (sea fish and alcohol) identified in Japanese studies [40, 79]. Lastly, the ‘Meat-based’ pattern (vegetables, root vegetables, mushrooms, seaweed, soybean products, potatoes, fruit, and a balanced intake of fish, meat, and dishes) was identified as a Japanese dietary pattern different from the ‘Dairy/vegetable-based’ pattern. This pattern was similar to the dietary pattern reported as the ‘Healthy Japanese’ pattern identified in a Japanese study [79]. These studies focused solely on food-related variables and did not consider other factors, such as dietary behavior and cooking methods. For example, the frequency of eating out [73], meal skipping [81], and the use of seasoning in cooking can all affect dietary patterns [82]. Simplifying dietary patterns can reduce related dietary behavior and cooking [83], but can also result in losing important information on dietary patterns. Therefore, it is vital to use a systematic approach in creating dietary patterns and consider food intake, dietary behavior, and cooking in the analysis [84–86]; this provides a more comprehensive understanding of the diet and helps identify specific dietary patterns.

In this study associations of ‘Dairy/vegetable-based’ and ‘Meat-based’ patterns with a lower risk of hypertension were observed. Our findings are consistent with previous studies revealing an inverse association between the risk of hypertension and the intake of beans, potatoes, mushrooms, vegetables, and healthy dietary behavior and healthy cooking [15, 16, 18, 19, 87], all of which were essential components of ‘Meat-based healthy’ dietary pattern identified in this study. A similar observation with a ‘Dairy/vegetable-based’ pattern was obtained in a large cohort study [88] demonstrating an association between ‘Vegetarian’ diet and a reduced risk of hypertension. The possible reasons for the reduced risk in ‘Dairy/vegetable-based’ dietary pattern may be in the lower intake of saturated fat, higher fiber consumption, and healthy dietary behavior. ‘Low-Fiber High-Sugar’ and ‘Seafood and Alcohol’ patterns were not specifically associated with the risk of hypertension. Previous reviews revealed similar results, indicating that compositions of diet patterns similar to our findings are associated with hypertension and other markers of metabolic dysfunction (blood pressure), with risk factors similar to those of hypertension [89]. Although marine products and vegetables have positive nutrient attributes, the patterns of other components, such as alcohol [90, 91], sweets [92, 93], and unhealthy dietary behavior (“eating speed” and “frequency of seasoning use in cooking”, etc.) [94, 95] related to higher risk of hypertension may have cancelled the benefit.

The BDHQ (Brief Self-Administered Diet History Questionnaire) employs a range of scales to represent the diverse and complex Japanese diet accurately. Varying scales are selected to capture subtle intake variations (e.g., a 7-point scale) or simpler scales are used when appropriate (e.g., a 5-point scale) [96]. This approach reflects the intricacies of assessing different foods, some straightforward and others requiring more detailed scales due to complexity or infrequent consumption. Researchers should be mindful of these scales when analyzing BDHQ data to maintain consistency and comparability in their findings, ensuring more comprehensive and reliable dietary research and nutritional assessment in Japan.

In this study, we did not include data from the year 2011 due to the potential impact of the Fukushima nuclear accident on the dietary habits of the study participants. This dietary change should be significant because nuclear contamination by accident [97] led to concern about food safety [96], which might affect hypertension development. Changes in the dietary patterns of the Fukushima nuclear accident would be an interesting topic, but it is beyond the current study. Similarly, another interesting issue would be the relationship between the dietary pattern change and the prevalence of other physical/mental disorders after the Fukushima nuclear accident. However, we have the health check-up data in 2011 but not the subsequent years, making it hard for longitudinal analysis. Therefore, we decided to focus on the relationship between dietary patterns and health by excluding 2011 data to avoid the potential impact of the Fukushima nuclear accident.

The results of the analysis in Table 3 (see Supplementary Table 3) indicate that food alone was not a strong predictor of incident hypertension. This was found using UMAP, K-means clustering algorithms, and multivariable logistic regression. This suggests that other factors, such as cooking methods and dietary behavior, may play a more important role in influencing the development of hypertension. It appears that the combination of foods, cooking methods, and dietary behaviors, as captured by the derived dietary patterns, gave a better prediction of the incidence of hypertension than the use of foods alone. This suggests that considering a wider range of dietary and lifestyle factors may provide a more accurate insight into the development of hypertension.

Additionally, it is essential to explore the possibility of 'white coat hypertension,' wherein blood pressure remains consistently elevated during out-of-office measurements but appears normal during in-office readings. This phenomenon may have implications for the interpretation of our results and should be considered in future research. To address this concern, we recommend that future studies explore methods for identifying patients at risk for white coat hypertension and utilize out-of-office readings for a more comprehensive assessment of participants' blood pressure. Furthermore, we encourage researchers to verify the validity of the blood pressure monitoring devices used in their studies.

Confounding factors are variables that can distort the relationship between exposure and outcome. In our study, we adjusted for confounding risk factors such as age, BMI, smoking, education, physical activity, dyslipidemia, diabetes, and salt intake and found an association between dietary patterns and hypertension. Salt intake was a potential mediator in our study, but the association between dietary patterns and hypertension persisted even after adjusting for salt intake, suggesting that other dietary factors may have contributed to the incidence of hypertension. This result is noteworthy because it suggests that there may be other dietary components, besides salt, that contribute to the incidence of hypertension. For instance, reducing salt intake alone was not associated with a reduction in blood pressure in the individuals with resistant hypertension [98, 99]. This fact implies that simply lowering salt intake may be insufficient to prevent hypertension and a more comprehensive approach that considers other dietary factors may be necessary [100]. Further research is needed to validate these findings and inform public health interventions to prevent hypertension.

Regarding hypertension, the diet based on Dietary Approaches to Stop Hypertension (DASH) has been shown to reduce blood pressure in previous studies [55, 56]. We estimated the DASH score using our dataset according to the procedure reported previously [55] (Supplementary Data 1) and compared with our results. In Table 1, the proportions of DASH scores ranging from Q1 to Q4 were presented for each cluster. The results revealed a noteworthy finding. We then analyzed the DASH scores for each cluster. Cluster D, a group with the highest risk of hypertension, showed the lowest DASH score. To evaluate the association between DASH scores and hypertension risk in Cluster D, we employed Cox proportional-hazards model. Our data showed relatively lower DASH scores compared with previous studies and no association with the incidence of hypertension (Table 2). This finding contrasts sharply with conclusions drawn previously using unsupervised machine learning methods. This suggests that within our dataset, unsupervised learning effectively identifies dietary patterns associated with the incidence of hypertension, possibly due to the presence of features related to dietary patterns that are well captured by unsupervised machine learning. However, when we shifted to using the DASH, we failed to observe similar associations in the data. This may indicate that the relationship between the DASH and the incidence of hypertension is not significant in our study sample, or alternatively, the DASH may not be a strong predictor in our dataset.

DASH scores from our dataset were relatively low compared with previous studies and showed no association with the incidence of hypertension (Supplementary Table 1), which contrasts sharply with the result using our unsupervised machine learning approach. This suggests that our machine learning approach could effectively identify dietary patterns associated with the incidence of hypertension even in the population with relatively low total DASH scores. Our result that there was no difference between quantile groups by DASH scores also suggests that DASH scores might overlook hypertension incidence when the contrast in the evaluation by DASH score is insufficient.

In the re-cluster with reduced categories, we observed that “only food items + dietary behaviors” could be effectively grouped into four clusters, while the other two sets, “Food items + cooking methods” and “Food items,” could only be categorized into two clusters. This suggests a critical contribution of dietary behaviors to clustering dietary patterns according to food items. Our study emphasizes the necessity of a multi-dimensional evaluation, including dietary behaviors and cooking methods in hypertension prevention.

Variable selection or aggregation of variables is often performed for providing more precise estimates, even though its effect has been controversial [104–106]. We also assessed the effect of grouping parameters on our clustering approach. We reclassified similar food items into each typical category, such as yogurt and milk, into the dairy product category. The grouping results are shown in Supplementary Data 1, providing two variables. Grouped dataset failed to produce clear clusters (Supplementary Fig. 6), possibly due to lower resolution by reduced parameter number.

However, our approach using unsupervised machine learning failed clustering by grouping similar food items (Supplementary Fig. 4). Dietary patterns are often complex and multifaceted, involving not only food types but also cooking methods and meal times [101]. Ignoring these factors can lead to oversimplification of dietary patterns, making it challenging to capture the true relationships within the data. By focusing solely on food items and neglecting dietary behaviors and cooking methods, essential dimensions of dietary patterns were omitted [102]. Different cooking methods can significantly alter the nutritional content and overall health impact of a meal, which is vital for understanding the complete picture of dietary habits [103]. The success of unsupervised machine learning techniques heavily depends on the quality and representativeness of the data [104]. If the dataset is incomplete, biased, or lacks diversity, the algorithm may struggle to identify meaningful patterns. It is crucial to ensure that the dataset used for clustering is comprehensive and accurately reflects the population's dietary habits.

Clustering using absolute and relative intake data failed to observe clear clusters in UMAP analyses as shown in Supplementary Figs. 7 and 8. The difference from our procedure using the BDHQ food intake frequency might be caused by absolute and relative intake “estimated” from the BDHQ frequency dataset with many assumptions, even though the previous study reported that the BDHQ showed reasonable validity regarding food intake estimates [43]. While our procedure used the frequency directly as a primary dataset with a simple conversion, the absolute intake and the total energy intake to calculate relative intake were estimated by “an ad hoc computer algorithm including weighting factors” (as described in the literature) which is not available in public [43], suggesting the estimated values by “black box” might make direct comparison difficult.

This study has several limitations. First, the self-reported method of dietary intake is susceptible to recall bias and social desirability bias, leading to the misclassification of food consumption and dietary behaviors. Although the validation of the BDHQ used in this study has been reported [43, 44, 105], the approach of asking about the frequency of food items consumed still has limitations in estimating the precise amount of nutrient intake [106, 107]. Additionally, the survey was conducted annually, and periodic changes were not considered. Furthermore, subjective bias can persist based on statistical thresholds or modeling selection, even in a data-driven approach. The other concern is that we hardly exclude the population and situation biases. Our participants, employees of Sendai Oroshisho Center, may not fully represent the diversity of Japanese males in terms of lifestyle, socio-economic backgrounds, and health behaviors. Our data also could not exclude the effect of “white coat hypertension” [108, 109], even though the blood pressure measurement was repeated when it showed higher values than medical standards. The measurement of the blood pressure by wearable devices or portable devices at home might be preferable to detect hypertension diagnosis more precisely. Lastly, it is crucial to note that in this study, we did not conduct a post hoc power analysis, which may impact the accuracy of interpreting and inferring observational effects. Future research should consider sample size and effect size during the design phase to ensure that the study has sufficient power. Another concern in the study is that the biased sample size (smaller sample size of Cluster A) might result in no difference compared to the reference (Cluster D) in the naïve analysis (Table 4, Models 2 and 3). Considering the possibility of over-adjustment, however, we performed a stepwise modeling through age-matched analyses and observed no difference for Cluster A in the age-matched ± 2 model (Table 6). This suggests that the confounding effect of age might be observed in Cluster A.

## Conclusion

Using unsupervised machine learning, UMAP and K-means clustering, on a cohort dataset of Japanese male population, we identified four dietary patterns: ‘Low-protein/fiber High-sugar,’ ‘Dairy/vegetable-based,’ ‘Meat-based,’ and ‘Seafood and Alcohol.’ ‘Seafood and Alcohol’ was identified as a risk dietary pattern with a highest prevalence of hypertension. The ‘Dairy/vegetable-based’ and ‘Meat-based’ showed a lower risk of hypertension incident compared to the ‘Seafood and Alcohol.’ Our study would be valuable for uncovering hidden patterns that traditional statistics and PCA approaches might overlook in nutritional research since our approach could consider food items, behavior, and cooking methods, offering insights into complex dietary patterns. Our holistic approach enhances understanding of how dietary choices impact the incidence of hypertension risk. Our study would provide a novel approach to identify specific patterns with potential implications for the incidence of hypertension.

### Supplementary Information

Below is the link to the electronic supplementary material.

Supplementary file1 (XLSM 43 kb)

Supplementary file2 (DOCX 133828 kb)

Supplementary file3 (DOCX 16 kb)

## Data Availability

The datasets generated during and/or analyzed during the current study are available from the corresponding author on reasonable request.
