# Supplementary material for: Dietary patterns associated with the incidence of hypertension among adult Japanese males: application of machine learning to a cohort study
Source: Eur J Nutr. 2024 Feb 25;63(4):1293–314. doi: 10.1007/s00394-024-03342-w (PMC11139695; doi:10.1007/s00394-024-03342-w)
Supplement: Supplementary file 3 — Supplementary file3 (DOCX 16 kb) [file 394_2024_3342_MOESM3_ESM.docx]

**Supplementary Tables**

| **Supplementary Table 1. HRs of hypertension According to Adherence to DASH Diet** | | | | | |
| --- | --- | --- | --- | --- | --- |
| Quantile of DASH Score | DASH Scores | HRs (95% CI)  Model 1 | P value | HRs (95% CI)  Model 2 | P value |
| Q 1 | 5-9 | Ref | 0.779 | Ref | 0.778 |
| Q 2 | 9-12 | 1.047 (0.818, 1.339) |  | 1.051 (0.821, 1.346) |  |
| Q 3 | 12-14 | 1.109 (0.836, 1.471) |  | 1.112 (0.838, 1.475) |  |
| Q 4 | 14-23 | 1.149 (0.866, 1.524) |  | 1.151 (0.865, 1.532) |  |
| Model 1: adjusted for age, BMI, smoking, education, and PA.  Model 2: Model 1 was additionally adjusted for dyslipidemia, diabetes, and salt intake. | | | | | |

| **Supplementary Table 2. The proportion of Q1~Q4 DASH score of each cluster.** | | | | |
| --- | --- | --- | --- | --- |
| Quantile | Cluster A (75) * | Cluster B (130) * | Cluster C (100) * | Cluster D (142) |
| Q 1 | 3 (4%) | 4 (3%) | 44 (44%) | 70 (49.3%) |
| Q 2 | 32 (42.7%) | 44 (33.8%) | 35(35%) | 35 (24.6%) |
| Q 3 | 22 (29.3%) | 32 (24.6%) | 13(13%) | 20 (14.1%) |
| Q 4 | 18 (24%) | 50 (38.5%) | 8 (8%) | 17 (12%) |
| Average of DASH Scores  (95% CI) | 13.11†  (12.52, 13.69) | 13.82†  (13.34, 14.29) | 10.38  (9.71, 11.05) | 10.35  (9.80, 10.89) |

* p < 0.0001. Statistical significance of quantile composition comparing Cluster D (Chi-square test).

† p < 0.0001. Statistical significance of average of DASH scores comparing Cluster D (Nonparametric comparison using Steel method).

| **Supplementary Table 3. Contribution of the category of parameters to clustering.** | | | | |
| --- | --- | --- | --- | --- |
|  | Cluster A | Cluster B | Cluster C | Cluster D |
| Food items + dietary behaviors + cooking methods (Original numbers) | 100% (75) | 100% (130) | 100% (100) | 100% (142) |
| Food items + dietary behaviors  (Overlap/numbers) | 90.7%  (68/83) | 91.5%  (119/136) | 79%  (79/86) | 88.7%  (126/146) |
| Food items + cooking methods | NA | | | |
| Food items | NA | | | |
| NA: not applicable. |  | | | |
