# Supplementary material for: Dietary patterns associated with the incidence of hypertension among adult Japanese males: application of machine learning to a cohort study
Source: Eur J Nutr. 2024 Feb 25;63(4):1293–314. doi: 10.1007/s00394-024-03342-w (PMC11139695; doi:10.1007/s00394-024-03342-w)
Supplement: Supplementary file 2 — Supplementary file2 (DOCX 133828 kb) [file 394_2024_3342_MOESM2_ESM.docx]

**Supplementary Figure 1.** Proportion of frequency score of the BDHQ dataset in each cluster. Bars represent a stacked proportion with colors corresponding to each BDHQ score.

A

A

B

C

D

B

**Supplementary Figure 2.** Clustering reliability by elbow (A) and silhouette (B) plots.

A. Elbow plot. The horizontal axis represents the number of clusters, and the vertical axis represents the within-cluster sum of squares (WCSS) centroid, which is the sum of the squared distances between each data point and its assigned cluster centroid. The optimal number of clusters determined by the Elbow method is 3 or 4. Cluster number 4 was chosen considering data point distribution in the scatter plot.

B. Silhouette plot. The cluster number 4 was confirmed reliable by the silhouette plot.

B

A

**Supplementary Figure 3.** Cluster plot (A) and Elbow plot (B) for clustering of “food items + dietary behaviors”. The scatterplot was overlaid with a density contour map.

A

B

**Supplementary Figure 4.** Cluster plot (A) and Elbow plot (B) for clustering of “food items + cooking”. The scatterplot was overlaid with a density contour map.

B

A

**Supplementary Figure 5.** Cluster plot for clustering of “dietary behaviors”. The scatterplot was overlaid with a density contour map.

A

B

**Supplementary Figure 6.** Cluster plot (A) and Elbow plot (B) for clustering of combined food items. The scatterplot was overlaid with a density contour map.

A

B

**Supplementary Figure 7.** Cluster plot (A) and Elbow plot (B) for clustering using absolute intake. The scatterplot was overlaid with a density contour map.

B

A

**Supplementary Figure 8.** Cluster plot (A) and Elbow plot (B) for clustering using relative intake. The scatterplot was overlaid with a density contour map.
